# Case Report: Pre- and postnatal management of an allantoic cyst with patent urachus and single umbilical artery

**DOI:** 10.12688/f1000research.6546.1

**Published:** 2015-05-22

**Authors:** Than Trong Thach, Vo Duy Quan, Tran Diem Nghi, Nguyen Hoang Anh, Le Phi Hung, Nguyen Thien Luan, Nguyen Phuoc Long

**Affiliations:** 1 University of Medicine and Pharmacy, Ho Chi Minh City, Ho Chi Minh, 70000, Vietnam; 2 Hung Vuong Hospital, Ho Chi Minh City, Ho Chi Minh, 70000, Vietnam; 3 School of Medicine, Vietnam National University, Ho Chi Minh City, Ho Chi Minh, 70000, Vietnam

**Keywords:** allantoic cyst, patent urachus, single umbilical artery

## Abstract

Patent urachus is a rare congenital abnormality. Since its first description by Cabriolus in 1550, few cases have been reported. A 26-year-old Vietnamese primigravida presented at 20 weeks of gestation for evaluation of a cystic mass in the umbilical cord, which was first discovered at week 13 of pregnancy by ultrasound scan. The cystic mass originated from the root of the umbilical cord, connected to the urinary bladder, and no intestinal contents were enclosed within. Doppler ultrasound assessment showed that the single umbilical artery existed within the normal range. The progression of the umbilical cyst continued to be screened, but the mass disappeared on ultrasound images at 27 weeks of gestation. This led to the consideration of the cyst’s rupture. After 38 gestational weeks, the pregnant woman delivered a 3350g male infant via cesarean section because of an obstructed vaginal labor. The following days, a stream of urine was recorded leaking out from the umbilical mass whenever he cried. Seven weeks after delivery, an open surgical approach was successfully performed. The baby is now 43 months of age, growing and developing normally. Since an allantoic cyst with patent urachus is a rare clinical entity, early discovery, close monitoring and accurate diagnosis through ultrasound in the prenatal period may consequently allow clinicians to have suitable attitudes towards management when the infant is born.

## Introduction

Urachus is a fibrous remnant of the allantois which communicates from the apex of the urinary bladder to the umbilicus. Failed obliteration of the urachus can lead to various abnormalities: urachal cyst, urachal diverticulum, sinus or patent urachus - the most common type ^1^.

Patent urachus, which was first described by Cabriolus in 1550, is an extremely rare clinical presentation, occurring in 1 to 2 or 2.5 per 100,000 deliveries ^2, 3^. Allantoic cysts in infants with patent urachus can be formed due to the drainage of urine into the umbilical cord ^4– 6^, or in uncommon situations, after leakage of hypo-osmotic urine into the Wharton’s jelly ^7, 8^.

Here we present a clinical case with a diagnosis of patent urachus. The newborn possessed a single artery in the umbilical cord and one allantoic cyst, and underwent a successful surgical resection.

## Case report

A 26-year-old Vietnamese primigravida, with no relevant medical, psychosocial or family history of morbidity, toxic exposure or abnormalities, after 1 year of marriage was informed of pregnancy at 7–8 weeks of gestation. The expected time for labor was October 1st 2011. First ultrasound screening at 12–13 weeks of gestation showed a 1.9mm nuchal translucency thickness. Simultaneously, a double test was also undertaken and demonstrated a trisomy 21 risk of 1/61000 and a trisomy 18 risk of 1/100000. Based on the fetal ultrasound exam, clinicians discovered a small cystic mass on the anterior abdominal wall whose nature remained unknown. No specific interventions were made and clinicians intended to keep observing the cyst. The next ultrasound exam at 16 weeks of gestation demonstrated a well-developing fetus with an enlarging cystic mass. However, its nature still remained unidentified due to lack of experience on image observation. Discrimination between allantoic cyst and pseudo-cyst presented a significant challenge for most of the ultrasonographists as well as clinicians at that time. Therefore, they decided to scrutinize it for one more month before deciding on a therapeutic intervention, especially since the triple test also showed low risk of trisomy 21, 18 and 13.

**Figure 1.**
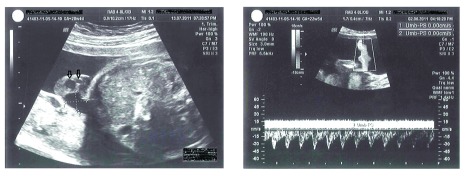
Traditional and Doppler ultrasound assessment showed only one umbilical artery (arrows).

**Figure 2.**
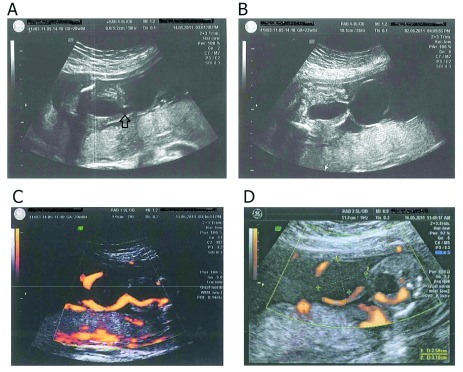
Umbilical cord cyst images. **A:** Umbilical cord cyst first detected at 20 0/7 weeks of gestation (arrow). **B:** Umbilical cord cyst was confirmed and a clear image of the connection between the bladder and the umbilicus was found at 22 5/7 weeks of gestation. **C:** Doppler velocimetry demonstrated flow around the cyst. **D:** Doppler ultrasound examined the size of the cyst.

The morphology ultrasound at 20 weeks of gestation on the fetus showed the presence of a single umbilical artery ( Figure 1) and a cystic mass (dimensions = 29mm × 25mm) at the root of the umbilical cord, connecting to the urinary bladder and no bowel contents enclosed within ( Figure 2A and Figure 2B). Doppler velocimetry revealed flows around this cyst ( Figure 2C). Other structures remained normal. Amniocentesis was indicated later and the result revealed a normal XY karyotype. A pediatric surgeon was invited to consult about this rare clinical entity and the consensus of postnatal operation for the neonate was finally made.

At 23 weeks of gestation, through the second morphology ultrasound, the cyst showed an increase in dimensions to 35mm × 28mm ( Figure 2D).

At 24 weeks of gestation, due to her weight gain reaching 13kg, an oral glucose tolerance test (OGTT) with 75g glucose was performed with a positive result for gestational diabetes. As a result, the patient was instructed to undertake a carbohydrate-restricted diet including 6 small meals plus 2 glasses of unsweetened milk every day. After 2 weeks, her blood glucose level returned to normal.

At 27 weeks of gestation, the cyst disappeared on ultrasound images, and a 180-minute echographic recording found no urine in the bladder. From the above findings, clinicians suspected that the cyst was ruptured, forming a fistula by which the urine refluxed into amniotic sac. No interventions were considered at this point. The next serial ultrasonography scan showed a little urine in the bladder but no umbilical cyst. Although there was only one single umbilical artery, the fetus still grew well, which was reflected by the growth chart of Hadlock with an estimated fetal weight (EFW) ranging from 70 ^th^–90 ^th^ percentile. A Doppler ultrasound evaluation using the Japan Society of Ultrasonics in Medicine (JSUM) 2001 proposal (More information can be found in the Voluson® E-Series (BT09) Advanced Reference Manual) showed that Resistive Index (RI) and Pulsatility Index (PI) of the umbilical artery and middle cerebral arteries was within the 20th and 50th percentile ^9^.

The patient had obstructed vaginal labor at 38 and 4/7 weeks of gestation with high suspicion of her limited pelvis. As a result, she underwent a cesarean section under general anesthesia and was administered Suxamethonium, Propofol, Esmeron and Sufentanil, after the failure of epidural block. Forty-five minutes later, the mother gave birth to a healthy male infant weighing 3350g. Post-delivery evaluation demonstrated a herniated sac-like mass (dimensions = 3mm × 4mm) at the root of umbilical cord and confirmed that the umbilical cord contained only one artery. The placenta was in normal condition. The umbilical cord was clamped about 5cm from the root.

In the following days, we recorded a stream of urine flushing out from the umbilical mass every time the infant cried. The umbilical tape was changed three times a day using normal saline and sterilized gauze. Five days after delivery, the umbilical stump dried and fell off, but the mass still persisted. The umbilical mass was still kept clean and covered by new tapes as described above until the operation at the infant’s seventh week. The open surgical approach was used under general anesthesia. Surgeons made a 2cm incision in the midline below the umbilicus, dissected rectus abdominis muscle and exposed the allantoic duct. They removed the duct, tied surgical knots using absorbable sutures, checked the two ureters and the bladder to ensure there were no further abnormalities and finally, the umbilicus was reconstructed. The patient was discharged 2 days after the operation without any complication.

At present, the patient is a 43-month-old boy with weight of 14kg, height of 100cm, and normal psychomotor development. He was re-checked several times at 3, 6 and 12 months of age, as well as every year after that with ultrasound examination and no abnormal structure was shown.

## Discussion

Urachal anomalies are the general name for several abnormal conditions (urachal cyst, sinus, patent urachus, or diverticulum) resulting from the failure of closing the allantoic duct during the 14 ^th^ gestational week ^3^. They are divided into two groups: congenital and acquired. Patent urachus, which accounts for 10–15% of all urachal remnant diseases, is usually congenital ^10^. Although newborns with congenital patent urachus have been recognized since the sixteenth century, prenatal diagnosis of this condition has only been carried out since 1988 ^2^ and our case is one of the first observed in Vietnam. As with other previous patent urachus cases, in our situation the mother underwent a morphology ultrasound examination at 20 weeks of gestation which revealed an extra-abdominal 29mm × 25mm cystoid structure near the umbilical cord root connecting to the fetal bladder. Omphalocele was ruled out because of the absence of fetal bowel contents within the channel. However, remarkably, only one umbilical artery was detected. Next ultrasonography performed at 27 weeks of gestation noticed the disappearance of the cyst. No urine was found in the fetal bladder after 180-minute recording. We suspected a rupture which created a fistula and drained the urine into the amniotic fluid. As the cyst ruptured in its early period, no umbilical cord compression was identified. The fetus developed normally regardless of the cyst and the presence of a two-vessel cord. At about 38 weeks of gestation, the pregnant woman was admitted with uterine contractions, and vaginal delivery was performed. Obstructed labor, however, subsequently took place, which prompted obstetricians to make an emergency cesarean section instead. To the best of our knowledge, only C. Rasteiro *et al.* has ever mentioned a case of allantoic cyst with patent urachus before ^11^. Unlike ours, in their description, although the cyst grew to 100mm in size, it did not burst and the amniotic fluid volume remained unchanged. Similarly, the bladder was empty but this was because the fetus micturated and urine leaked into the cyst. Although there have been several patent urachus cases reported, our patent urachus case with a ruptured cyst in the setting of single-umbilical-artery fetus is unique.

A cystoid mass perceived through ultrasound may be involved in different conditions, which makes the prenatal diagnosis questionable ^7^. Particularly, differentiation between a pseudo- and a true cyst can regularly confound ultrasound diagnosis, despite the fact that very rare cases have been distinguished based on ultrasound imaging. Nevertheless, there are at least three features which can give us important suggestions. A true cyst is always in close contact with the fetal anterior abdominal wall, surrounded by umbilical vessels which can be verified by color flow imaging, and shows an open channel from the cyst to the bladder and the fetal umbilicus ^12^. The gold standard for differential diagnosis between a pseudo- and a true cyst is histopathology. Under the microscope, a true cyst comprises an epithelial lining and originates from remnants of the allantoic duct while the pseudo-cyst is the result of the degeneration or the edema of Wharton’s jelly ^12^. A pseudo-cyst not only makes up a higher incidence but is also more associated with aneuploidy and other chromosomal disorders than a true cyst ^13^. Hence, obstetricians are usually recommended to perform a karyotype once they discover a cystic lesion. In our current case, we assumed that it was indeed a true cyst. A karyotype was executed but no peculiarities were found. This poses another complicated problem - the best and earliest time for authenticating a true cyst. In medical literature, Waldo Sepulveda *et al.* reported three cases of patent urachus detection in the first trimester at 11 to 14 weeks’ gestation, based on the findings of megacystis and the large umbilical cord cyst ^6^. In most of the cases, patent urachus was recognized during the second and third trimester. However, clinicians still remained unconfirmed about this condition until the urine dribbles from the umbilicus right after the delivery, and our case was no exception ^6^.

The recognition of patent urachus is not easy due to a very low frequency of 1- 2.5:100000 live births ^2, 3^ and non-specific signs and symptoms. Therefore, specialists should be accustomed to it and be prepared for the treatment. Different methods can be used to manage this condition. Complete surgical removal of the urachus is one of the general recommendations with favorable outcome ^14^. Surgical techniques can avoid malignant conversion and other complications occurring together with increasing age whereas conservative treatment (i.e. drainage and antibiotic therapy) sometimes leads to recurrent infection or cyst formation. Apropos of surgical treatment, experts and researchers up to now have still debated on the optimal method and have not come to any conclusion. Prior to the endoscopic era, open excision was the main method for removing the urachus. A large ten-year retrospective study focusing on open surgery conducted by Mesrobian *et al.* in 1997 did not show any complications or reoperations afterward ^15^. Open surgery also required shorter hospital stays ^16^. However, since the appearance and universal application of laparoscopy in 1995 ^17^, several studies have suggested that this method could be a safe and effective alternative with minimal morbidity ^18^. Laparoscopic approach not only provides an all-around visualization of the urachal channel and fetal bladder but also improves patient’s general state and reduces post-operative pain more rapidly, as do all endoscopic procedures ^16^. In our case presented here, surgeons and urologists who participated in that operation opted for open excision. The best time to execute a patent urachus surgical removal also remains controversial ^19^.

An open surgical approach was carried out when the neonate was seven weeks old. The operation ended with success and the male baby was discharged after two days. He was observed closely over the following days and no further complications were found. The baby grew up normally. At present, he is a 43-month-old boy with physical and psychomotor development corresponding to his age. Ultrasound examination has again verified his normal anatomical structure. Through this case, we are likely to make a conclusion that patients with isolated patent urachus who already underwent a surgical removal may have a far more optimistic prognosis. Medical literature describes several early-treated patent urachus neonates who then developed well with no anomalies ^14, 20^, which might support our hypothesis. However, no optimal follow-up time interval was accurately proposed. In the past, Sepulveda *et al.* reported a case with a three-year follow-up, wherein no abnormalities were ultimately revealed ^6^. Ideally, a randomized, prospective study with larger population should be conducted to achieve a certain conclusion.

Our case demonstrates some limitations. We could have diagnosed patent urachus earlier but in reality, we could not reach the confirmation till 20 weeks of gestation. Our lack of experience may be the major reason, since it was the first time we encountered this urachal remnant disease in Vietnam. Regardless of the inevitable limitation detailed above, the main aim of our paper is to present a new case and provide more information about patent urachus, hence contribute partly to a better and safer treatment of this rare congenital anomaly.

## Conclusion

Briefly, we have described a ruptured allantoic cyst with patent urachus in the setting of single umbilical artery fetus. The majority of patent urachus were discovered in the second and third trimester. Regarding our case, open resection may also achieve optimistic prognosis without any complication, hospital readmission and reoperation. In conclusion, there are still challenges on turning out early diagnosis and better treatment methods.

## Consent

Informed consent for publication of images and information from this case was obtained from the mother of the infant.
