# Supplementary material for: Case Report: Pre- and postnatal management of an allantoic cyst with patent urachus and single umbilical artery
Source: F1000Res. 2015 May 22;4:124. [Version 1] doi: 10.12688/f1000research.6546.1 (PMC4505787; doi:10.12688/f1000research.6546.1)
Supplement: Peer review history

# Peer review: Referee response for version 1

**DOI:** 10.5256/f1000research.7029.r9460

**Published:** 2015-07-13

**Authors:** Gamal Serour

**Affiliations:** 1 Department of Obstetrics and Gynaecology, Al Azhar University, Cairo, Egypt

This is a case report of a very rare congenital anomaly. Its indexation would be of interest to obstetricians and gynaecologists as it outlines methods of diagnosis and treatment.

I have read this submission. I believe that I have an appropriate level of expertise to confirm that it is of an acceptable scientific standard.

# Peer review: Referee response for version 1

**DOI:** 10.5256/f1000research.7029.r8742

**Published:** 2015-05-27

**Authors:** John Svigos

**Affiliations:** 1 Maternal Fetal Medicine Unit, Women’s and Children’s Hospital, Adelaide, SA, Australia

The article is well written and descriptive of the likely problems encountered with this rare condition.

Whilst the problem was corrected at open surgery, laparoscopic surgical excision represents the contemporary management with its reduced post operative morbidity and length of hospital stay although both methods are equally successful in correcting the defect.

I have read this submission. I believe that I have an appropriate level of expertise to confirm that it is of an acceptable scientific standard.
